# Timing of elective pre‐labour caesarean section: A decision analysis

**DOI:** 10.1111/ajo.12821

**Published:** 2018-04-26

**Authors:** Freke A. Wilmink, Clarabelle T. Pham, Nicole Edge, Chantal W.P.M. Hukkelhoven, Eric A.P. Steegers, Ben W. Mol

**Affiliations:** ^1^ Department of Obstetrics and Gynaecology Radboudumc Nijmegen The Netherlands; ^2^ School of Public Health University of Adelaide Adelaide South Australia Australia; ^3^ Department of Obstetrics and Gynaecology Mildura Base Hospital Mildura Victoria Australia; ^4^ Perined Utrecht The Netherlands; ^5^ Department of Obstetrics and Gynaecology Erasmus MC – Sophia Children's Hospital Rotterdam The Netherlands; ^6^ Department of Obstetrics and Gynaecology The Robinson Research Institute University of Adelaide North Adelaide South Australia Australia

**Keywords:** caesarean section, decision analysis, neonatal respiratory morbidity, respiratory distress syndrome, transient tachypnoea of the newborn

## Abstract

**Background:**

Since caesarean sections (CSs) before 39^+0^ weeks gestation are associated with higher rates of neonatal respiratory morbidity, it is recommended to delay elective CSs until 39^+0^ weeks. However, this bears the risk of earlier spontaneous labour resulting in unplanned CSs, which has workforce and resource implications, specifically in smaller obstetric units.

**Aim:**

To assess, in a policy of elective CSs from 39^+0^ weeks onward, the number of unplanned CSs to prevent one neonate with respiratory complications, as compared to early elective CS.

**Materials and Methods:**

We performed a decision analysis comparing early term elective CS at 37^+0–6^ or 38^+0–6^ weeks to elective prelabour CS, without strict medical indication, at 39^+0–6^ weeks, with earlier unplanned CS, in women with uncomplicated singleton pregnancies. We used literature data to calculate the number of unplanned CSs necessary to prevent one neonate with respiratory morbidity.

**Results:**

Planning all elective CSs at 39^+0–6^ weeks required 10.9 unplanned CSs to prevent one neonate with respiratory morbidity, compared to planning all elective CSs at 38^+0–6^ weeks. Compared to planning all elective CSs at 37^+0–6^ weeks we needed to perform 3.3 unplanned CSs to prevent one neonate with respiratory morbidity.

**Conclusion:**

In a policy of planning all elective pre‐labour CSs from 39^+0^ weeks of gestation onward, between three and 11 unplanned CSs have to be performed to prevent one neonate with respiratory morbidity. Therefore, in our opinion, fear of early term labour and workforce disutility is no argument for scheduling elective CSs <39^+0^ weeks.

## Introduction

Rates of caesarean sections (CSs) are increasing in Australia with 33.4% of women birthing by CS in 2015, compared to 28.5% in 2003.^1^, ^2^ Of all birthing women in 2015, 21% underwent CS prior to labour.^2^ This has significant health implications for infants, as elective CSs are associated with higher rates of neonatal respiratory morbidity compared to intended vaginal delivery.^3^, ^4^, ^5^ As this discrepancy reduces with increasing gestational age until 39^+0^ weeks of gestation, clinical guidelines in Australia and New Zealand, the UK and the USA recommend delaying elective pre‐labour CS until 39^+0^ weeks gestation.^5^, ^6^, ^7^, ^8^, ^9^, ^10^, ^11^, ^12^, ^13^ Alternative recommendations are verification of lung maturity or to administer corticosteroids in advance for those CSs planned prior to 39^+0^ weeks.^7^, ^11^, ^14^ In 2012 still 51.6% of pre‐labour CS occurred before 39^+0^ weeks in Australia.^2^ This percentage seems even higher in private hospitals (66.8%).^15^ On one hand, delay of elective CSs until 39 weeks bears the risk of earlier spontaneous labour resulting in an unplanned CS. Given that in Australia more than 25% of women give birth in regional or remote areas, this may have resource and workforce implications as in these locations staff are often not on‐site continuously and an unplanned CS may require initiation of ‘call‐backs’.^1^ On the other hand, these locations are more likely to have limited access to neonatal facilities and thus reducing the potential for neonatal complications is crucially important. Use of a decision analysis tree is a quantitative approach to clinical problem solving that utilises local, national and international data to estimate the probabilities of a certain outcome. In this decision analysis we assessed, in a policy of elective CSs from 39^+0^ weeks onward, the number of unplanned CS that would be required to prevent one infant with respiratory complications.

## Materials and Methods

### Definitions of CS

An elective (pre‐labour) CS is defined as a planned CS without strict medical indication and performed before the start of labour. An unplanned CS is defined as a CS performed before the scheduled date after start of labour.

### Decision tree model

**Figure 1:**
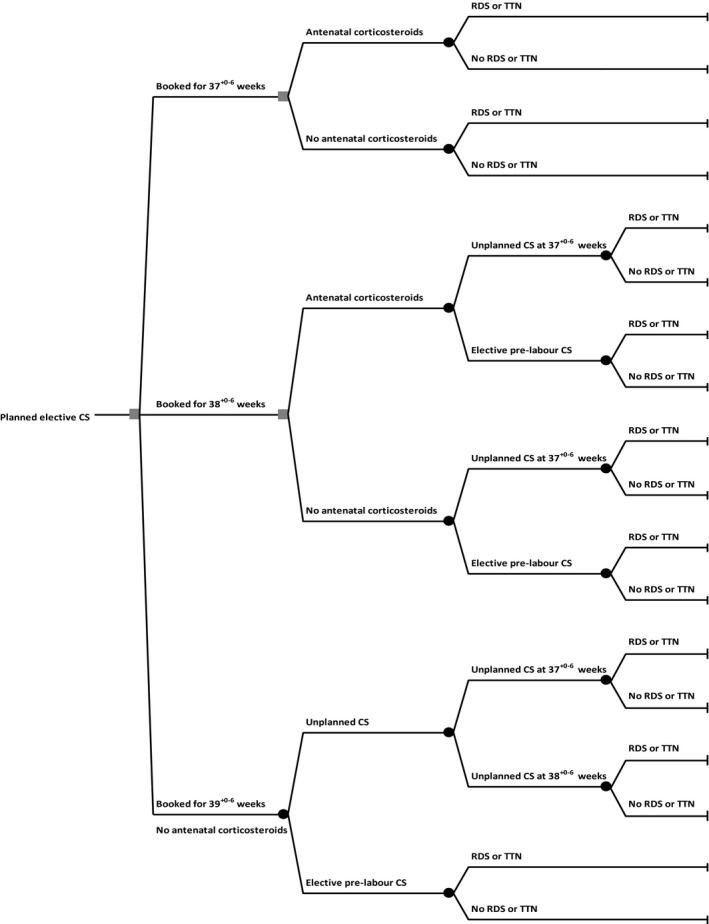
Decision tree. CS, caesarean section; RDS, respiratory distress syndrome; TTN, transient tachypnoea of the newborn

Decision tree modelling enables the comparison of the outcomes of alternative clinical strategies in the absence of clinical trials.^16^, ^17^ A decision tree with a three‐week time horizon was constructed to model possible clinical strategies for timing of birth for women requiring elective CS (Fig. 1). The decision tree begins with three main branches stemming from a decision node, which represents the choice of a clinical strategy. A series of branches from each of the clinical strategies represents the different paths and outcomes for particular combinations of events. Each path on the decision tree has a probability of being taken and an outcome (neonate with or without respiratory morbidity). The sum of the main outcome from the series of branches for each strategy was then calculated and compared. We assumed that elective CSs are not planned before 37^+0^ weeks of gestation. The three main strategies, *without* the administration of antenatal corticosteroids, were:

1. elective CS planned at 39^+0–6^ weeks of gestation, with risk of an unplanned CS at 37^+0^ until 38^+6^ weeks of gestation
2. elective CS booked at 38^+0–6^ weeks of gestation, with risk of an unplanned CS between 37^+0–6^ weeks of gestation
3. elective CS booked at 37^+0–6^ weeks of gestation.

Alternative strategies, *with* the administration of antenatal corticosteroids, were:

1. elective CS booked at 38^+0–6^ weeks of gestation, with risk of an unplanned CS at 37^+0–6^ weeks of gestation
2. elective CS booked at 37^+0–6^ weeks of gestation.

A second decision tree was constructed to include the possibility of lung maturity testing, using probabilities based on the sensitivity and specificity of the Lecithin‐Sphingomyelin (L/S) ratio test (figure not shown).

An alternative strategy with performing an L/S ratio test was:

1. elective CS booked at 37^+0–6^ weeks of gestation after performing an L/S ratio test.

Our main outcome measure was the number of unplanned CSs needed to prevent one neonate with respiratory morbidity defined as respiratory distress syndrome (RDS) or transient tachypnoea of the newborn (TTN).

### Data sources

The probabilities that were used as inputs for the decision tree model were based on previous literature and pregnancy outcome data from state‐based and national government publications.^2^, ^18^ To obtain relevant data we searched PubMed: ‘Caesarean Section’ [Mesh] in combination with the following free search terms: ‘elective’ or ‘respiratory morbidity’ or ‘corticosteroids’. In addition, our search was filtered by ‘English’ and ‘last 10 years’. The definitive search was performed on 9 July, 2017 and produced 1786 hits. After scanning titles, abstracts and cross‐references we identified five cohort studies and one Cochrane review which presented data about neonatal respiratory morbidity following term elective CS stratified by week of gestation from 37^+0^ weeks of gestation onward (Table 1).^5^, ^8^, ^9^, ^10^, ^19^, ^20^ The rate of unplanned CSs is based on the rate of singleton live births with spontaneous start of labour, with all women still having to deliver as the denominator, in 2012 until 2014. This was 3.75% between 37^+0^ and 37^+6^ weeks and 12.39% between 37^+0^ and 39^+0^ weeks of gestation, which corresponds with data from international literature.^8^, ^10^, ^20^

**Table 1:** Neonatal respiratory morbidity associated with elective caesarean section

Reference	*n*	Respiratory outcome	Weeks		
			37^+0–6^ % (*n*/*n*_total_)	38^+0–6^ % (*n*/*n*_total_)	39^+0–6^ % (*n*/*n*_total_)
Morrison *et al*.^10^	2341	NICU admission with RDS or TTN	7.4 (27/366)	4.2 (45/1063)	1.8 (9/505)
Hansen *et al*.^5^	32 580	Combined respiratory morbidity^a^	10.0 (20/191)	5.1 (55/1083)	2.1 (22/1051)
Tita *et al*.^9^	13 258	RDS or TTN	8.2 (68/833)	5.5 (213/3904)	3.5 (221/6510)
		RDS	3.7 (31/833)	1.9 (75/3904)	0.9 (58/6510)
		TTN	4.8 (40/833)	3.9 (153/3904)	2.7 (178/6510)
Wilmink *et al*.^8^	20 973	Combined respiratory morbidity^b^	6.8 (118/1734)	3.5 (356/10 139)	2.1 (136/6647)
		RDS or TTN	4.6 (80/1734)	2.7 (277/10 139)	1.5 (98/6647)
		RDS	0.5 (8/1734)	0.2 (21/10 139)	0.1 (7/6647)
		TTN	4.3 (74/1734)	2.5 (256/10 139)	1.4 (91/6647)
Chiossi *et al*.^19^	23 749	RDS or TTN	9.1 (118/1296)	6.4 (294/4601)	4.0 (274/6941)
		RDS	3.8 (49/1296)	2.1 (98/4601)	1.0 (71/6941)
		TTN	5.3 (69/1296)	4.3 (196/4601)	2.9 (203/6941)

NICU, neonatal intensive care unit; RDS, respiratory distress syndrome; TTN, transient tachypnoea of the newborn.

a ICD‐10 codes including any respiratory distress, TTN, or persistent pulmonary hypertension.

b RDS, TTN, pneumothorax, oxygen supplementation, intermittent positive pressure ventilation or continuous positive airway pressure.

### Analysis

Following the decision tree the number of unplanned CSs needed to be performed to prevent one neonate with respiratory morbidity was calculated, based on the obtained data. The risks of delivering earlier with an unplanned CS were taken into account. We assumed incidence rates of respiratory morbidity in neonates born after unplanned CS to be equivalent to the incidence rates of respiratory morbidity after elective CS.

### Sensitivity analyses

One‐way sensitivity analyses were performed to address the uncertainty and examine the impact of the probabilities, at 37^+0–6^, 38^+0–6^ and 39^+0–6^ weeks of gestation, of respiratory morbidity and unplanned CS on the cost to prevent one neonate with respiratory morbidity for our four main strategies. The lower and upper 95% limits were tested for the following parameters.

Probability of respiratory morbidity with:

- *elective* CS at 37^+0–6^ weeks *with* and *without* antenatal corticosteroids
- *unplanned* CS at 37^+0–6^ weeks *with* and *without* antenatal corticosteroids
- *elective* CS at 38^+0–6^ weeks *with* and *without* antenatal corticosteroids
- *unplanned* CS at 38^+0–6^ **weeks *without* antenatal corticosteroids
- *elective* CS at 39^+0–6^ weeks *without* antenatal corticosteroids.

Probability of having an *unplanned* CS at:

- 37^+0–6^ weeks with a *planned elective* CS at 38^+0–6^ weeks
- 37^+0–6^ or 38^+0–6^ weeks with a *planned elective* CS at 39^+0–6^ weeks.

This study was exempt from institutional review board ethical approval.

## Results

### Number of unplanned CSs needed to perform

Pooled incidences of RDS and TTN were calculated per week of gestation with separate outcome data from three large cohort studies.^8^, ^9^, ^19^ Pooled incidences of RDS and TTN together were 6.9%, 4.2% and 2.9% for neonates born at 37^+0–6^, 38^+0–6^ or 39^+0–6^ weeks of gestation, respectively (Table 2). This corresponds with a risk reduction of 39.1% by planning at 39^+0–6^ weeks compared to 38^+0–6^ weeks and of 58.0% by planning at 39^+0–6^ weeks compared to 37^+0–6^ weeks of gestation. We used data from the Cochrane review to calculate the risk reduction of respiratory morbidity after elective CS *with* the administration of antenatal corticosteroids. These relative risks (95% confidence intervals) were 0.49 (0.16‐1.57) and 0.44 (0.17‐1.14) at 37^+0–6^ and 38^+0–6^ weeks, respectively.^20^

**Table 2:** Pooled incidences of RDS and TTN associated with elective caesarean section

Reference	*n*	Respiratory outcome	Weeks		
			37^+0–6^%	38^+0–6^%	39^+0–6^%
Pooled incidences^a^	44 539	RDS or TTN	6.9	4.2	2.9
		RDS	2.3	1.0	0.7
		TTN	4.7	3.3	2.4

RDS, respiratory distress syndrome; TTN, transient tachypnea of the newborn.

a Pooled incidences with separate outcome data from Tita *et al*.,^9^ Wilmink *et al*.^8^ and Choissi *et al*.^19.^

In Table 3 we present the results of our decision analysis.

**Table 3:** Number of unplanned caesarean sections needed to be performed to prevent one neonate with RDS or TTN

Elective CS booked (weeks)	Number of emergency CS needed to perform^a^
Strategy *without* administration of antenatal corticosteroids	
For 39^+0–6^ compared to 38^+0–6^	10.9
For 39^+0–6^ compared to 37^+0–6^	3.3
For 38^+0–6^ compared to 37^+0–6^	1.4
Strategy *with* administration of antenatal corticosteroids	
For 38^+0–6^ *with* AC compared to 37^+0–6^ *with* AC	2.5
For 38^+0–6^ *with* AC compared to 39^+0–6^ *without* AC	3.0
Strategy *without* administration of antenatal corticosteroids after a *positive* L/S *ratio*	
For 39^+0–6^ compared to 37^+0–6^	3.9

AC, antenatal corticosteroids; CS, caesarean section; RDS, respiratory distress syndrome; TTN, transient tachypnoea of the newborn.

a Number of emergency CS needed to perform to prevent one neonate with RDS or TTN.

Main strategies *without* the administration of antenatal corticosteroids:

1 when planning all elective CSs at 39^+0–6^ weeks, to prevent one neonate with respiratory morbidity we needed to perform:
  - 10.9 unplanned CSs, compared to planning all elective CSs at 38^+0–6^ weeks.
  - 3.3 unplanned CSs, compared to planning all elective CSs at 37^+0–6^ weeks.
2 when planning all elective CSs at 38^+0–6^ weeks, to prevent one neonate with respiratory morbidity we needed to perform 1.4 unplanned CSs, compared to planning all elective CS at 37^+0–6^.

Strategies *with* the administration of antenatal corticosteroids:

1. when planning all elective CSs at 38^+0–6^ weeks *with* antenatal corticosteroids, to prevent one neonate with respiratory morbidity we needed to perform:
  - 2.5 unplanned CSs, compared to planning all elective CS at 37^+0–6^ *with* antenatal corticosteroids
  - 3.0 unplanned CSs, compared to planning all elective CS at 39^+0^ weeks *without* antenatal corticosteroids.

Strategy with performing an L/S ratio test:

1. when planning all elective CSs at 39 weeks, to prevent one neonate with respiratory morbidity we needed to perform 3.9 unplanned CSs compared to planning all elective CSs at 37^+0–6^ weeks after a positive L/S ratio test (and therefore assuming sufficient lung maturity).

## Discussion

### Main findings

With a policy change of booking all elective CSs at 39^+0–6^ weeks of gestation, depending on prior booking policy at 37^+0–6^ or at 38^+0–6^ weeks of gestation, between three and 11 unplanned CSs are necessary to prevent one neonate with respiratory morbidity, respectively. Correspondingly, this will reduce the risk of a neonate with respiratory morbidity by approximately 50%. If delaying until 39^+0^ weeks is absolutely not possible because of fetal or maternal complications, a strategy with the administration of antenatal corticosteroids and booking an elective CS at 38^+0–6^ weeks compared to booking an elective CS at 37^+0–6^ weeks of gestation, 2.5 unplanned CSs need to be performed to prevent one neonate with respiratory morbidity. To prevent one neonate with respiratory morbidity, 3.9 unplanned CSs need to be performed if delaying until 39^+0^ weeks of gestation, compared to delivery at 37^+0–6^ weeks after a positive L/S ratio test (indicates fetal lung immaturity).

### Strength and limitations

Our decision analysis shows valid and clear results and can assist in common practice and informed clinical decision‐making. Large randomised trials are lacking and absolute numbers of severe respiratory morbidity at term are low. Therefore, we used data of large observational studies, published in peer‐reviewed journals. All showed the same decreasing trend of respiratory morbidity with an increase of gestational age from 37^+0^ to 39^+0^ weeks onward, assuming validity. There were some uncertainties. We had no data on the incidence of respiratory morbidity in neonates born with unplanned CS after onset of labour; therefore we assumed these incidences to be equal, in agreement with recent literature.^21^ If this assumption is not correct (literature shows conflicting results),^10^ and neonatal respiratory outcome after an intrapartum CS would be better compared to neonatal respiratory outcome after a prelabour CS, the number of CS needed to perform to prevent one sick neonate would be even lower. If the incidences of neonatal respiratory morbidity in CSs after the onset of labour were lower than after a planned elective CS, the numbers of unplanned CSs to perform to prevent one neonate with respiratory complications would be even lower. As numbers were too small to have valid data on the incidence of RDS and TTN, risk reduction of respiratory morbidity after administration of antenatal corticosteroids was calculated based on ‘admission to a special baby care unit with respiratory morbidity’.^20^ Although there are several adverse neonatal outcome measures associated with early term elective CSs (hypoglycaemia, hyperbilirubinaemia, sepsis, longer hospitalisation and neonatal intensive care admission),^8^ we only assessed respiratory morbidity as a neonatal outcome measure, as this is the most important cause of neonatal morbidity in early term births.

### Interpretation

The number of unplanned CSs needing to perform will be translated into increased unplanned workforce and will have resource implications, but not necessarily only out of office hours. In a recent trial only 34% with a planning strategy at 39^+3^ and 36% with a planning strategy at 38^+3^ weeks of unplanned CSs was out of hours.^22^ One could argue that besides resource implications, unplanned CSs might have higher maternal risks compared with elective CSs. However, a large analysis did not show this.^23^ In addition to respiratory morbidity, in CSs before 39^+0^ weeks, the risk for hypoglycaemia and longer neonatal admission is also significantly higher, causing maternal‐neonatal separation which is associated with reduced rates of breast‐feeding initiation.^8^, ^9^, ^24^ Also postnatal transfer to a more equipped neonatal unit can be necessary. In addition, a growing body of evidence suggests that children born early term have a greater risk for developmental delay in the first two years of life, have higher chances of respiratory morbidity like wheezing and asthma and more often have special educational needs.^25^, ^26^, ^27^, ^28^, ^29^

### Antenatal corticosteroids

A commonly used alternative strategy is administration of antenatal corticosteroids to all women with a planned CS before 39^+0^ weeks. A randomised clinical trial comparing all women undergoing a CS at term with and without prior administration of antenatal corticosteroids did show a decrease of admittance to special baby care units, (relative risk 0.46, 95% CI: 0.23–0.93); however, this decrease was not separately significant for TTN or RDS.^14^, ^20^ Follow up in childhood did not show less asthma in the treated group and school assessment showed children in the treated group to be significantly more often in the lowest quartile of academic ability.^30^ Furthermore, a recent trial showed an increase of neonatal hypoglycaemia in late preterm infants in the treated group (24.0% vs 15.0%) which has been associated with impaired neurologic outcome in childhood.^31^, ^32^ As the incidence of respiratory morbidity at term is low, following a strategy with administration of antenatal corticosteroids to all mothers, more than 95% of mothers and their neonates will be unnecessarily exposed to possible long‐term risks.

Lung maturity tests as the L/S ratio (sensitivity 74.6% and specificity of 82.5%) and only performing a CS when the result indicates mature lungs as an alternative is questionable. Bates *et al*. reported that early‐term born infants, despite adequate fetal lung maturity with L/S ratio test, still had a significant higher risk of TTN compared to infants born at 39–40 weeks gestation.^33^ Quantus, a new non‐invasive lung maturity test, unfortunately has no better test performances (sensitivity 62.1%, specificity 91.3%) between 34^+0^ and 38^+6^ weeks.^34^

### Future research

We recommend a large prospective cohort study planning all elective CSs at or beyond 39^+0^ weeks of gestation, to assess both neonatal and maternal outcome of elective and all necessary unplanned CSs in the short and long term.

## Conclusion

In a policy of planning all elective pre‐labour CSs from 39^+0^ weeks of gestation onwards, between three and 11 unplanned CSs have to be performed to prevent one neonate with respiratory morbidity. Therefore, in our opinion, fear of early‐term labour and workforce disutility is no argument for scheduling elective CSs <39 weeks. Further justification might be provided by conducting a comprehensive economic evaluation. Administration of corticosteroids instead of postponing an elective CS until after 39^+0^ weeks is no good alternative until more data about long‐term consequences are available.
